# Novel glycopolymer sensitizes *Burkholderia cepacia* complex isolates from cystic fibrosis patients to tobramycin and meropenem

**DOI:** 10.1371/journal.pone.0179776

**Published:** 2017-06-29

**Authors:** Vidya P. Narayanaswamy, Scott Giatpaiboon, Shenda M. Baker, William P. Wiesmann, John J. LiPuma, Stacy M. Townsend

**Affiliations:** 1 Synedgen, Inc., Claremont, California, United States of America; 2 University of Michigan, Department of Pediatrics and Communicable Diseases, Ann Arbor, Michigan, United States of America; Laurentian, CANADA

## Abstract

*Burkholderia cepacia* complex (Bcc) infection, associated with cystic fibrosis (CF) is intrinsically multidrug resistant to antibiotic treatment making eradication from the CF lung virtually impossible. Infection with Bcc leads to a rapid decline in lung function and is often a contraindication for lung transplant, significantly influencing morbidity and mortality associated with CF disease. Standard treatment frequently involves antibiotic combination therapy. However, no formal strategy has been adopted in clinical practice to guide successful eradication. A new class of direct-acting, large molecule polycationic glycopolymers, derivatives of a natural polysaccharide poly-N-acetyl-glucosamine (PAAG), are in development as an alternative to traditional antibiotic strategies. During treatment, PAAG rapidly targets the anionic structural composition of bacterial outer membranes. PAAG was observed to permeabilize bacterial membranes upon contact to facilitate potentiation of antibiotic activity. Three-dimensional checkerboard synergy analyses were used to test the susceptibility of eight Bcc strains (seven CF clinical isolates) to antibiotic combinations with PAAG or ceftazidime. Potentiation of tobramycin and meropenem activity was observed in combination with 8–128 μg/mL PAAG. Treatment with PAAG reduced the minimum inhibitory concentration (MIC) of tobramycin and meropenem below their clinical sensitivity breakpoints (≤4 μg/mL), demonstrating the ability of PAAG to sensitize antibiotic resistant Bcc clinical isolates. Fractional inhibitory concentration (FIC) calculations showed PAAG was able to significantly potentiate antibacterial synergy with these antibiotics toward all Bcc species tested. These preliminary studies suggest PAAG facilitates a broad synergistic activity that may result in more positive therapeutic outcomes and supports further development of safe, polycationic glycopolymers for inhaled combination antibiotic therapy, particularly for CF-associated Bcc infections.

## Introduction

*Burkholderia cepacia* complex (Bcc) is a group of genotypically diverse strains currently consisting of 20 species and are the causative agent of severe lower respiratory infections in cystic fibrosis patients [1]. Colonization of the lung with Bcc is associated with an increased risk of accelerated pulmonary decline, early death, and often excludes Bcc infected patients from consideration for lung transplantation [2, 3]. Furthermore, Bcc respiratory infections in CF patients frequently lead to exacerbations, causing a significant and rapid decline in lung function that is often not reversible [4].

Bcc have the ability to resist and adapt to antibiotic treatment and adverse environmental conditions, making it virtually impossible to eradicate from the CF lung [5–7]. Antimicrobial therapies for infection caused by Bcc are severely limited by the broad-spectrum resistance exhibited by most strains [8, 9]. Meropenem and tobramycin are two commonly used antimicrobial agents that are generally recommended to treat CF pulmonary exacerbation [10], however only a few reports describing treatments for CF patients infected with Bcc have been published [4, 11–14]. Though these antimicrobial agents cause a reduction in bacterial density, clinical improvement in lung function was not observed [11]. Opportunities exist for developing new, more effective therapeutic strategies potentially involving the use of multiple antibiotic therapies to treat chronic lung infections associated with CF.

Development of alternate antibacterial strategies to potentiate the antimicrobial activity of conventional antibiotics have become increasingly important due to the emerging threat of multi-drug resistant infection. Poly (acetyl, arginyl) glucosamine (PAAG), is a recently discovered novel class of glycopolymer therapeutics that demonstrate broad antibacterial activity across a spectrum of drug resistant antibiotics and in many cases, has shown synergy with antibiotics *in vitro* [14]. A wide range of pathogenic bacteria associated with CF disease, including methicillin-resistant *Staphylococcus aureus* (MRSA), *Pseudomonas aeruginosa*, and nontuberculous mycobacteria (NTM), are sensitive to PAAG alone or in combination with antibiotics [15]. PAAG is a polycationic polysaccharide and is observed to be biocompatible with minimal eukaryotic cytotoxicity [16]. Many antimicrobial peptides and polyethylenimines (PEI) are also polycationic, but have some degree of cytotoxicity that limits their use [14]. Rapid permeabilization of bacterial membranes facilitated by the affinity of PAAG for anionic structures has been previously observed [17]. Due to the physiochemical properties of polysaccharides, their derivatives have been widely used in food [18–21] and therapeutic industries [22–26] without significant risk. Safety and tolerability studies conducted on PAAG, demonstrate a lack of toxicity upon inhalation and intravenous administration in the animal experiment studies.

PAAG and similar cationic antimicrobials such as polymyxin B interact with the outer membrane of gram-negative bacteria depolarizing the membrane resulting in leakage of the intracellular contents and death. This mode of action facilitates increased resistance characterized by a lack of charged molecules ability to cross the membrane and exert antimicrobial effects. Specifically, divalent cations located within the bacterial outer membrane support the integrity of the structure and electrostatically link lipopolysaccharide (LPS) by the anionic phosphate groups [27, 28]. Gram-negative bacteria were shown to be susceptible to the antimicrobial activity of PAAG, and antibacterial activity of PAAG was enhanced by increased charge density [29]. Tobramycin is a polycationic glycoside that facilitates antibacterial activity by penetrating the bacterial membrane and attaching to the 30S and 50S ribosome and preventing protein translation [30]. Similarly, meropenem is a cationic antibiotic that inhibits synthesis of the bacterial cell wall [31]. Ceftazidime is an anionic antibiotic that also penetrates cells and binds to proteins essential for cell elongation facilitating the bactericidal activity. Diffusion through the bacterial cell wall is required to facilitate the antibacterial activity of each of these antibacterial agents [32]. The different antibacterial targets could facilitate the combination effect of these drugs in treating Bcc.

This study examines the scope of synergy and sensitization among seven Bcc clinical isolates from CF patients and one environmental isolate using 3D checkerboard analysis to comparatively examine antibiotic combinations of meropenem (MEM) and tobramycin (TOB) with PAAG compared to those with ceftazidime (CAZ). The results infer that the combination therapy with PAAG might be effective to treat lung infections caused by Bcc in CF patients. This study infers that combination therapy with PAAG is effective to treat lung infections caused by Bcc in CF patients. The bactericidal activity of PAAG may help to clear bacteria from lungs, thereby decreasing bacterial sputum density, which may help improve clinical symptoms.

## Materials and methods

### Bacterial strains and culture conditions

Eight Bcc isolates were examined in this study. One isolate was obtained from ATCC (ATCC 25416). Six isolates were obtained from the *Burkholderia cepacia* Research Laboratory and Repository (BcRLR) (University of Michigan, Ann Arbor, MI) of which four (AU10321, AU10529, AU8042, and AU10398) were the subject of previously reported studies evaluating novel antimicrobial agents [8]. One isolate used for this study (EH4) was newly isolated from a CF sputum sample provided from the Gregory Fleming James Cystic Fibrosis Research Center in Birmingham, Alabama [Dr. Steve Rowe], by techniques described below.

The sputum sample was processed by liquefaction in 1% dithiothreitol (DTT) as described previously [33]. Dilutions (1:1) were made and plated onto selective media. Isolates with distinctive colony morphologies were sub cultured for further characterization by conventional methods [34]. The DNA from the Bcc isolate designated EH4 was sent to AccuGENIX (Charles River Laboratories, Inc.) and identified and typed via comparative 16S ribosomal RNA (rRNA) gene sequence and reported as *Burkholderia multivorans*. All the bacterial cultures were maintained as frozen stocks at -80°C in Mueller-Hinton (MH) broth with 15% glycerol and recovered from frozen stock on MH agar following overnight incubation at 37°C.

### Antimicrobial agents and susceptibility testing

Synedgen's polycationic proprietary glycopolymer is an arginine derivative of a natural polysaccharide poly-N-acetyl-glucosamine (PAAG). It is polycationic and soluble at physiologic pH's. The antibiotics meropenem and tobramycin were obtained from Sigma-Aldrich (St. Louis, MO). Each of the *Burkholderia* isolates were tested against meropenem (0.375–48 μg/mL), tobramycin (2–1024 μg/mL), ceftazidime (8–1024 μg/mL) and PAAG (8–1024 μg/mL). Susceptibility testing was performed by a broth microdilution method with the test antibiotics and PAAG under test conditions in accordance with Clinical and Laboratory Standards Institute (CLSI) standards. Serial two-fold dilutions of the test preparation namely meropenem, tobramycin, ceftazidime and PAAG were made in supplemented MH broth and 100 μl was aliquoted into each well of 96-well flat bottom microtiter plates. Bacteria grown overnight in MH broth were diluted to a 1 McFarland turbidity standard in MH broth. The diluted culture was added to the wells being tested. Bacteria without the addition of antibiotics or PAAG were used as controls. The plates were incubated at 37°C for 24h. The isolates were categorized as sensitive, intermediate, or resistant according to the CLSI guidelines [35].

### Checkerboard assay

**Fig 1:**
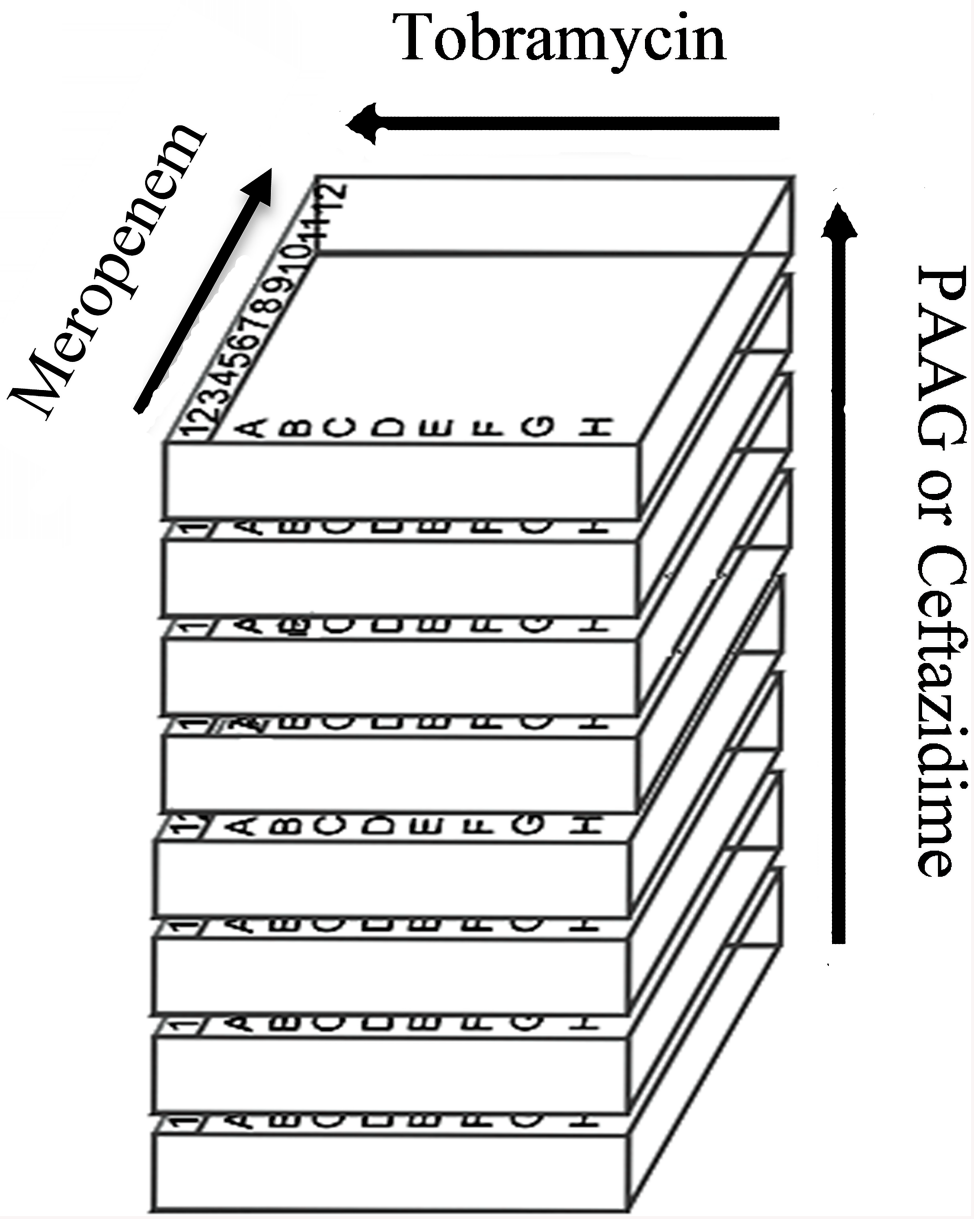
Schematic of the set-up for a three-dimensional checkerboard technique. Diagram depicts the design of the 96-well test plates to include 3 different antibiotics. Each plate has a fixed concentration of PAAG or ceftazidime. Each plate also has a standard checkerboard of meropenem vs tobramycin. The arrows indicate increasing concentrations of each antimicrobial drug. The diagram is adapted from Stein *et*. *al*., 2015 [38].

The eight isolates of *Burkholderia* were characterized as resistant based on the MICs of the selected individual antibiotics using the microdilution method [36, 37]. The MICs were determined using three-dimensional checkerboard microdilution assay with MH Broth (Difco) and a final inoculum of 5 X 10^7^ CFU/mL. Microdilution of triple antibiotic combinations in the presence and absence of PAAG were performed in the following manner as depicted in Fig 1. The checkerboard assays were performed in duplicates (n = 2). The control plate contained increasing concentrations of meropenem (48–0.375 μg/mL) on the x-axis and increasing concentration of tobramycin (2–1024 μg/mL) on the y-axis. Each of the subsequent eight plates contained fixed concentrations of PAAG ranging from 2–1024 μg/mL with increasing concentration of tobramycin ranging from 2–1024 μg/mL on the x-axis and increasing concentrations of meropenem ranging from 0.375–48 μg/mL on the y-axis. MICs and fractional inhibitory concentrations (FIC’s) were determined after a period of 24h growth. The MIC was defined as the lowest concentration well in the microtiter plate which had no visible growth in it. The FICI values < 0.5, 1.0, and >4 were defined as synergistic, additive or indifferent, and antagonistic respectively, according to the previously published methods [38].

## Results

### Antimicrobial susceptibility of Bcc isolates

The clinical isolates examined were taken from patients having a mean age of 24.1 years (3 males, 4 females), and were isolated in the years between 1995 and 2016, from various geographic regions across the United States (data not shown). Antibiotic susceptibility for meropenem, tobramycin, and ceftazidime was determined for all the isolates used in the study. The minimum inhibitory concentration (MIC) for each of the isolates against these antibiotics is listed in Table 1. Table 1 also summarizes the results of the three-dimensional checkerboard study and documents the observed relationships as determined by fractional inhibitory concentration (FIC) index (FICI). PAAG alone showed antibacterial activity against all the Bcc strains tested at concentrations exceeding 1024 μg/ml.

**Table 1:** *In vitro* activities of combination antibiotic treatment and PAAG.

Species Tested	Strains Tested	MIC(μg/mL)				FICI_MEM/TOB_	FICI_MEM/TOB/CAZ_	FICI_MEM/TOB/PAAG_
		MEM	TOB	CAZ	PAAG			
*B*. *multivorans*	AU10398	6	512	32	>1024	0.39 ± 0.12 (S)	4.1 ± 0.22 (NS)	0.14 ± 0.11 (S)
*B*. *cenocepacia*	AU10321	24	128	>1024	>1024	0.42 ± 0.23 (S)	0.3 ± 0.10 (S)	0.15 ± 0.21 (S)
*B*. *multivorans*	AU2380	24	256	>1024	>1024	1.0 ± 0.32 (NS)	4.25 ± 0.24 (NS)	0.1 ± 0.14 (S)
*B*. *multivorans*	AU0064	24	256	32	>1024	0.8 ± 0.14 (NS)	4.1 ± 0.23 (NS)	0.1 ± 0.12 (S)
*B*. *cenocepacia*	AU0007	24	256	128	>1024	0.16 ± 0.16 (S)	0.4 ± 0.14 (S)	0.1 ± 0.13 (S)
*B*. *cenocepacia*	AU0037	12	128	8	>1024	0.3 ± 0.13 (S)	1.31 ± 0.14 (NS)	0.15 ± 0.21 (S)
*B*. *cepacia*	ATCC 25416	6	256	128	>1024	0.05 ± 0.21 (S)	1.5 ± 0.12 (NS)	0.12 ± 0.14 (S)
*B*. *multivorans*	EH4	6	256	64	>1024	0.8 ± 0.12 (NS)	2.5 ±0.14 (NS)	0.15 ± 0.14 (S)

Data is represented as the FICI values ± standard deviation. FICI values given are the lowest values for the given combination.

S = Synergy, NS = No synergy, MEM = Meropenem, CAZ = Ceftazidime, TOB = Tobramycin, FICI = Fractional inhibitory concentration index, MIC = Minimum inhibitory concentration.

### Synergy testing

Eight isolates were found to be clinically resistant to meropenem, tobramycin, and ceftazidime were tested further in combination with PAAG, using the three-dimensional checkerboard microdilution assay. The MICs of for the 8 isolates used for three-dimensional checkerboard analysis were between 6–24 μg/mL for meropenem, between 8 and >1024 μg/mL for ceftazidime, and 128–512 μg/mL for tobramycin and all isolates exhibited resistance to all three antibiotics, according to the CLSI standard [35]. The MICs of the individual MICs of each antibiotic have been detailed in Table 1 to demonstrate individual drug activity with respect to the FICI values. *In vitro* antibiotic combinations are characterized based on the FICI, which represents the sum of the FICs of each drug tested. The FIC of each drug combination is determined by dividing the MIC of each drug when used in combination by the MIC of each drug when used alone (FIC A/B/C = MIC of A (combination)/ MIC of A alone + MIC of B (combination)/ MIC of B alone + MIC of C (combination) / MIC of C alone. The FICI interpretation used was FICI < 0.5 synergy, 0.5 < FICI < 4 additive effects or indifference, and FICI ≥ 4 antagonism [36, 37, 39, 40].

All 8 isolates tested with MEM/TOB/PAAG treatments demonstrated synergistic relationships demonstrated by an FICI of less than 0.5. For each isolate tested in combination with PAAG, the MIC_MEM_ and MIC_TOB_ was reduced 16 to 64-fold (Table 1). Modest synergy demonstrated by a 4-fold reduction in the MIC_CAZ_ was also observed with MEM/TOB/CAZ treatments against *B*. *cenocepacia* AU10321 and AU0007 (Table 1). All other isolates showed either no favorable interaction between the drugs or antagonistic relationships. The isolates that demonstrated antagonism for MEM/TOB/CAZ antibiotic treatment (*B*. *multivorans* strains AU10398, AU2380, and AU0064), showed increased MIC_CAZ_ values in the presence of meropenem and tobramycin.

**Fig 2:**
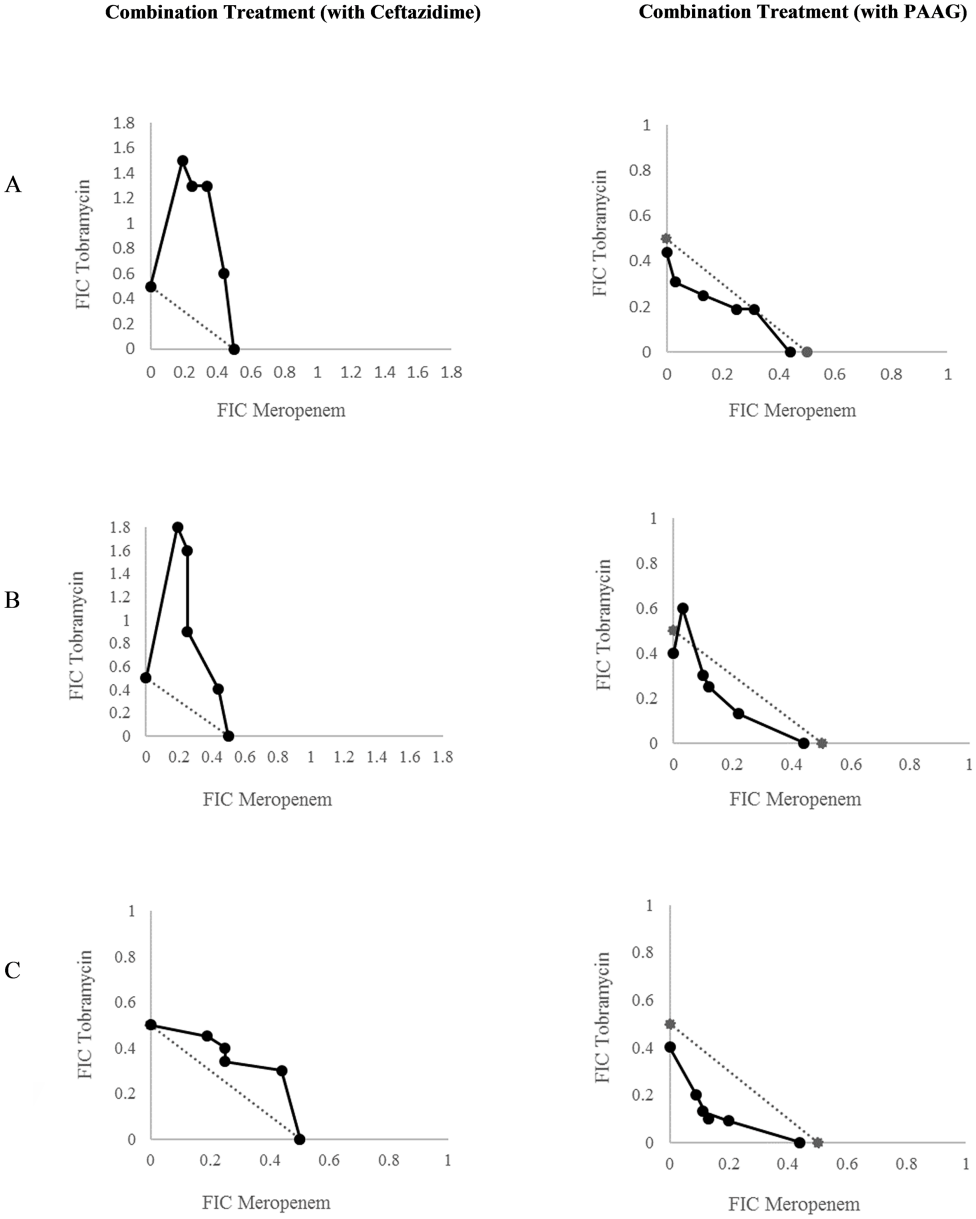
Isobole analysis of synergistic antibiotic activity. (A) *B*. *multivorans* EH4, (B) *B*. *multivorans* AU10398, (C) *B*. *cenocepacia* AU10321. The lowest FIC index values of the combinations were reported as median +/- standard deviation. The graphs on the right column shows isoboles of Bcc strains exhibiting synergistic effects with the triple combination antibiotic treatment of meropenem, tobramycin and PAAG. The graphs on the left column exhibits isoboles of Bcc strains with a triple combination antibiotic treatment of meropenem, tobramycin and ceftazidime. The FIC values were chosen from the lowest concentration of antibiotics where addition of PAAG that could potentiate the effect of the antibiotics. Dotted line refers to FIC 0.5.

In the presence of PAAG -concentrations as low as 8 μg/mL, all Bcc strains tested showed 2 to >16 fold reductions in the minimal concentration that inhibited growth (MIC) of meropenem and tobramycin. Lack of significant synergy was observed when PAAG concentrations below 8 μg/mL was used for the three-dimensional checkerboard assay. More strains will be tested for synergy at lower concentrations of PAAG. Combination therapy with antibiotics (other than ceftazidime), in the presence of PAAG, showed a more pronounced reduction in the MIC for all 8 Bcc clinical isolates tested (Table 1). The 2D checkerboard assays used as control, in the absence of PAAG, also demonstrated synergistic relationships for most of the strains tested. The therapeutic antibiotic combination of MEM/TOB/CAZ is frequently used in clinical practice to treat CF lung infections caused by Bcc. Treatment with PAAG facilitated synergy at the lowest concentrations of MEM and TOB tested (2 μg/mL of TOB and 0.375 μg/mL of MEM) (Fig 2). PAAG sensitizes resistant bacteria below clinical sensitivity breakpoints for all the eight clinical isolates of Bcc tested. Ceftazidime was unable to reduce tobramycin or meropenem MIC of *B*. *multivorans* EH4 below clinical breakpoints *in vitro*. However, replacing ceftazidime with PAAG (8–128 μg/mL) sensitizes *B*. *multivorans* EH4 to meropenem. The MIC_TOB_ for *B*. *multivorans* EH4 was reduced 32-fold in the presence of PAAG and meropenem. Although achieving an MIC_TOB_ of 2 μg/mL for *B*. *multivorans* EH4 was below the sensitivity breakpoint [34]. Isolate *B*. *multivorans* AU10398 and *B*. *cenocepacia* AU10321 were both re-sensitized to meropenem and tobramycin with the addition of PAAG. Four other isolates demonstrated similar results of which these represent. These studies show that PAAG sensitizes Bcc to lower MICs of meropenem and tobramycin compared to use with ceftazidime in combination therapy.

Isoboles were used to graphically represent the synergistic relationships between antibiotics and PAAG. The FIC values evaluated in Fig 2 were derived from the lowest concentration of antibiotics where addition of PAAG was able to potentiate the effect of the antibiotics. In Fig 2, values below the broken line specifies FICs below 0.5 and indicate synergistic relationships and values above the broken line demonstrate a lack of synergy between the tested antibiotics. The left column of Fig 2 exhibits isoboles of Bcc strains treated with meropenem, tobramycin and ceftazidime. The right column exhibits isoboles of Bcc strains exhibiting synergistic effects with a triple combination of meropenem, tobramycin and PAAG. Specifically, Fig 2 shows the isoboles of *B*. *multivorans* EH4 (Fig 2A), *B*. *multivorans* AU10398 (Fig 2B), and *B*. *cenocepacia* AU10321 (Fig 2C) demonstrating PAAG’s superior synergistic activity in combination with meropenem and tobramycin, compared to ceftazidime. Four other CF clinical isolates demonstrated similar results (S1 Fig) and speaks to the reproducibility and robustness of the observation of PAAGs superior activity. These data show that FIC values of antibiotic combinations including PAAG achieve synergy at lower concentrations than antibiotic combinations with ceftazidime, and suggest a more rapid and efficient *in vitro* antibacterial activity can be achieved with the use of PAAG.

## Discussion

The current study served to characterize the interactions of PAAG in combination with meropenem and tobramycin as a novel therapeutic strategy against highly resistant bacterial infections. Multi-drug resistant Bcc were sensitized to antibiotic treatment by PAAG (Table 1). Tobramycin (TOB), meropenem (MEM), and ceftazidime (CAZ) are frequently used in combination therapy for Bcc infection and yet, were insufficient to reduce tobramycin or meropenem effective concentrations below clinical antibacterial sensitivity breakpoints (meropenem ≤ 4 μg/mL, tobramycin ≤ 4μg/mL) *in vitro* (Fig 2). Antibiotic synergy was observed at lower tobramycin and meropenem concentrations when used in combination treatment with PAAG (2 μg/mL of TOB and 0.375 μg/mL of MEM) compared to ceftazidime (64–128 μg/mL of TOB and 6–12 μg/mL of MEM) and demonstrates the ability of PAAG (compared to ceftazidime) to reduce MIC of tobramycin and meropenem, below CLSI breakpoints. Also, combination antimicrobial treatment that included PAAG resulted in synergy for all the clinical isolates tested, compared to only two *B*. *cenocepacia* isolates treated in combination with ceftazidime (Table 1). In fact, antagonistic relationships were observed in three of the four *B*. *multivorans* isolates when treated with ceftazidime, tobramycin, and meropenem. This observation underlines the importance of recognizing the limitations in predictive value of *in vitro* antibiotic susceptibility testing when using small numbers of representative bacterial species tested, as well as variables associated with host and testing methodology [41].

Among Bcc lung infections associated with cystic fibrosis, *B*. *cenocepacia* and *B*. *multivorans* represent the most commonly found strains [42, 43]. The significance of the different antibiotic susceptibilities of bacterial species has not been extensively examined in previous synergy studies [44]. The current observation of antagonism in *B*. *multivorans* treated with ceftazidime, tobramycin, and meropenem, compared to the achievement of synergy in *B*. *cenocepacia* clinical isolates (Table 1), supports the claim that significant variation exists in antibiotic susceptibility among *Burkholderia* species. Consequently, the observed antagonism between these cationic antibiotics and anionic ceftazidime observed when used against *B*. *multivorans* could be dependent on the differences in charge and their interaction with outer membrane structure [9]. Observations by others show 18% of ceftazidime-sensitive isolates demonstrated antagonistic relationships with the addition of tobramycin [11]. Also, 13% of tobramycin-sensitive strains and 5% of meropenem-sensitive strains demonstrated antagonism in the presence of ceftazidime [11]. The epidemiology and diversity of Bcc species may explain this observation of antagonism of single antibiotic sensitive Bcc isolates [1, 11]. In comparison, treatment of these strains with PAAG in combination with meropenem and tobramycin achieved synergy against both species equally. Further investigation of treatment regimens with regard to specification of Bcc species isolated is warranted, specifically with respect to *B*. *cenocepacia* and *B*. *multivorans* that make up a majority of Bcc infections [1]. Failure to account for the differences between *Burkholderia* species may result in less favorable clinical outcomes and impede the development of appropriate treatment recommendations.

Consensus has not been met on the appropriate treatment regimen for Bcc lung infections, despite numerous studies examining antibiotic combination therapy [4, 11–13]. The differences in resistances between strains may make such a broad consensus impossible. Both multi-drug resistance and genetic diversity among Bcc species complicate clinical treatment. Studies demonstrate therapeutic success against Bcc when the patient is treated with tobramycin, meropenem, and/or ceftazidime [6, 44]. The drawbacks of these standard antibiotic treatments include increased resistance development, antagonism, and adverse effects. PAAG, and other potentiating glycopolymer, may provide an appealing drug development strategy by augmenting the activity of ineffective or inefficient therapeutic agents and demonstrating synergistic activity over a broad range of Bcc species. PAAG, as a polycationic antimicrobial that selectively permeabilizes bacterial membranes appears to facilitate antimicrobial activity. Bacterial membrane permeability was increased following PAAG treatment as demonstrated by bacterial uptake of florescent probes (1-N-phenylnaphthylamine, nile red, and propidium iodide) and supported by electron microscopy documentation of abnormal outer membrane structure [29]. Other cationic small-molecules attempt to utilize this strategy to potentiate antibacterial activity however, the required concentrations were too high for clinical use given the toxicity of the drugs [27]. The current study did not elucidate mechanisms of antibiotic resistance but suggests patterns in antibiotic susceptibility profiles that may inform current treatment practices.

Antibiotics such as polymixin B, colistin, and colistimethate sodium (CMS) are associated with harmful side-effects (i.e., nephrotoxicity, ototoxicity, and neuromuscular blockade) and are limited in clinical use in favor of treatments with better safety profiles [45]. For example, CMS can be used as an inhaled treatment for chronic lung infections but requires relatively large doses (up to 160 mg/kg every 8 hours) and CMS is associated with nephrotoxicity at a rate similar to the more potent polymixin B [45, 46]. Ongoing studies suggest that less than 1mg/kg will be safely tolerated and effective for PAAG treatments delivered by inhalation at less frequent intervals than what is standard for CMS [45]. Further, as large-molecule therapeutics, glycopolymer PAAG is restricted to the local treatment area and is unable to cross into the circulation to exhibit systemic toxicity and also may reduce the effective dose. Cytotoxicity was evaluated in the elution, agar diffusion, and direct contact tests and indicated PAAG was non-toxic. Acute dermal irritation and delayed-type hypersensitivity tests reported no irritation or sensitivity to PAAG treatment. In preliminary nonclinical toxicology studies, PAAG has been administered intravenously without any major adverse clinical signs at more than 1000 times or 100 times the anticipated therapeutic dose in rats and dogs, respectively. Dose escalation studies of inhaled PAAG for 7-days showed no treatment related clinical signs nor any test article-related effects observed in either rats or dogs. These data show a feasible and safe dose of PAAG is achievable and encourages further efforts in the development of PAAG for treatment of CF airway infections.

These preliminary results presented in this study suggest that triple antibiotic combination therapy with PAAG is more effective and bactericidal against clinical Bcc isolates, when compared to the standard triple antibiotic combination therapy with ceftazidime. PAAG has been shown to enhance susceptibility of resistant Bcc during combination antibacterial treatment. Triple synergy assay utilizing antibiotic combination therapy with PAAG is currently being further evaluated with more Bcc species and other isolates associated with CF infections. Further pre-clinical and clinical studies are ongoing to investigate the use of PAAG as a novel inhaled therapeutic to potentiate and restore susceptibility of innately resistant Bcc infections and improve clinical outcomes.

## Supporting information

S1 Fig Isoboles of the double combinations of the antibiotics. (A) *Burkholderia multivorans* AU2380, (B) *Burkholderia multivorans* AU0064, (C) *Burkholderia cenocepacia* AU0007.
(PDF)
