# Supplementary material for: Novel glycopolymer sensitizes Burkholderia cepacia complex isolates from cystic fibrosis patients to tobramycin and meropenem
Source: PLoS One. 2017 Jun 29;12(6):e0179776. doi: 10.1371/journal.pone.0179776 (PMC5491046; doi:10.1371/journal.pone.0179776)

Combination Treatment (with Ceftazidime) **Combination Treatment (with PAAG)**

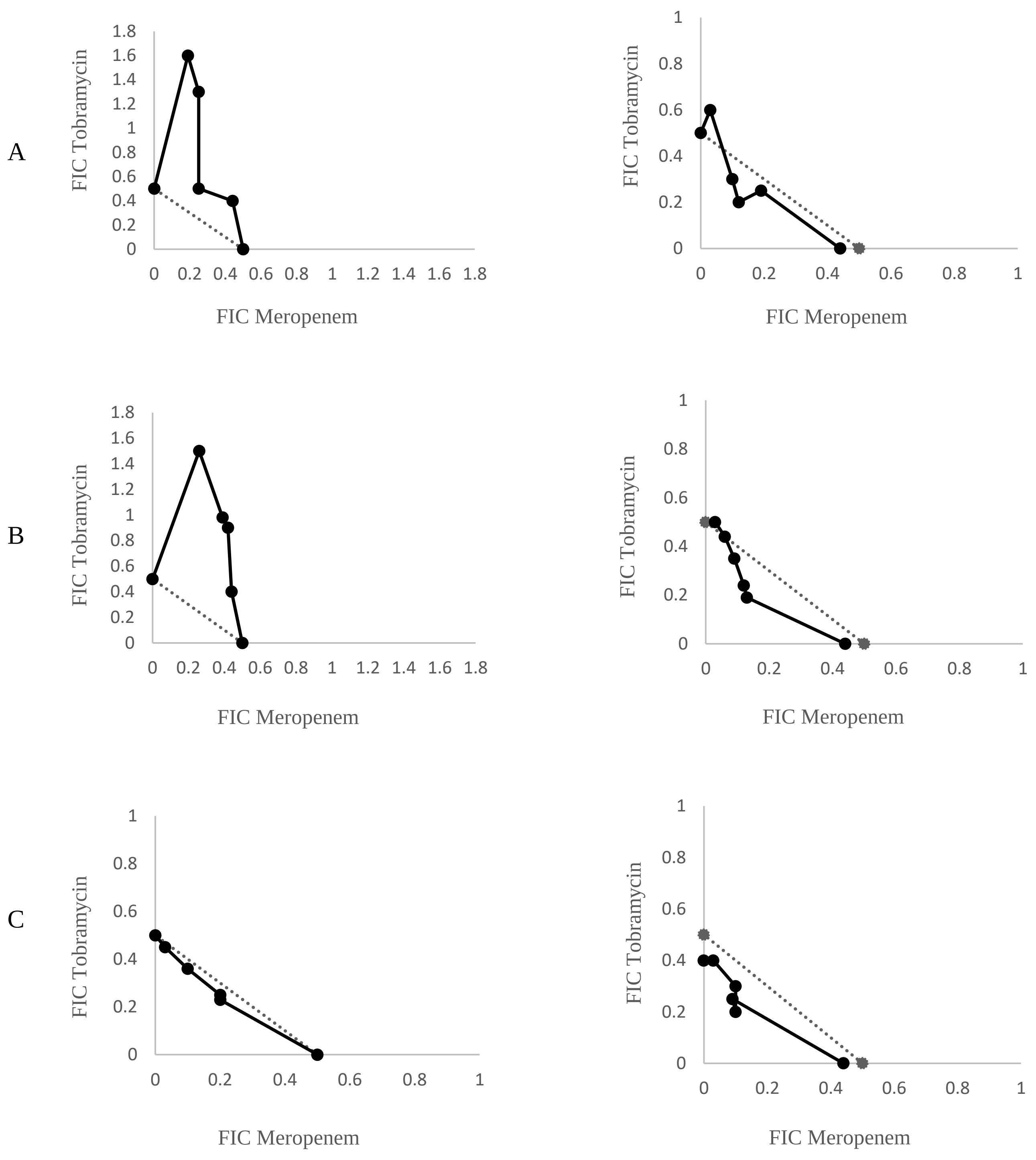

Combination Treatment (with Ceftazidime)

Combination Treatment (with PAAG)

D

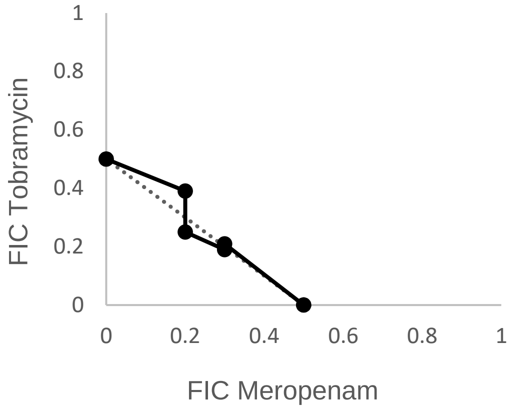

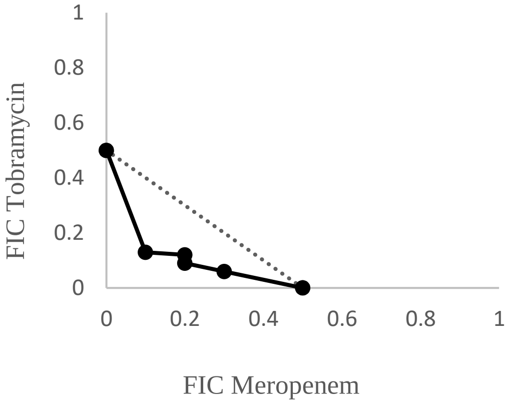

E

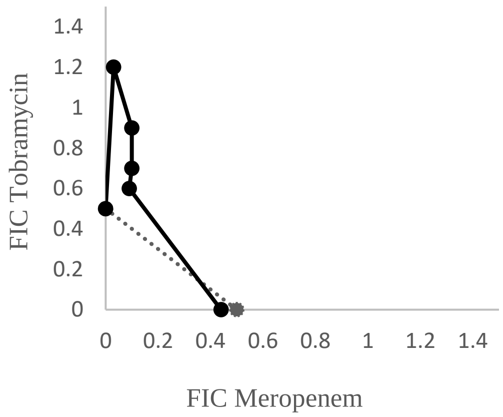

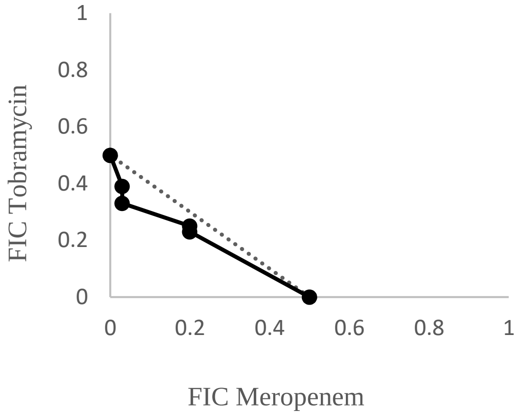

Supplement: S1 Fig — (A) Burkholderia multivorans AU2380, (B) Burkholderia multivorans AU0064, (C) Burkholderia cenocepacia AU0007. (PDF) [file pone.0179776.s001.pdf]
